# Social Capital during the First Wave of the COVID-19 Outbreak: The Case of the Island of Menorca

**DOI:** 10.3390/ijerph182312720

**Published:** 2021-12-02

**Authors:** Ester Villalonga-Olives, Ichiro Kawachi, Ildefonso Hernández-Aguado

**Affiliations:** 1 Pharmaceutical Health Services Research Department, University of Maryland School of Pharmacy, Baltimore, MD 21201, USA; 2 Department of Social and Behavioral Sciences, Harvard T.H. Chan School of Public Health, Boston, MA 02115, USA; ikawachi@hsph.harvard.edu; 3 Department of Public Health, History of Science and Gynecology, Miguel Hernández University and CIBER de Epidemilogía y Salud Pública, 03550 Alicante, Spain; ihernandez@umh.es

**Keywords:** social capital, COVID-19, qualitative research, epidemiology, public health

## Abstract

The rapidly evolving coronavirus pandemic has drastically altered the economic and social lives of people throughout the world. Our overall goal is to understand the mechanisms through which social capital shaped the community response to the pandemic on the island of Menorca, Spain, which was under a strict lockdown in 2020. Between April and June 2020, we performed qualitative interviews (*n* = 25) of permanent residents of the island. From the findings, it is evident that social capital is an important resource with the capacity to organize help and support. However, the dark sides of social capital, with lack of social cohesion and lack of trust, also emerged as an important negative issue. We identified sources of tension that were not resolved, as well as important sociodemographic differences that are primary drivers for health inequalities. The investment in social networks and social capital is a long-term need that should consider sociodemographic vulnerability.

## 1. Introduction

The rapidly evolving coronavirus pandemic has drastically altered the economic and social lives of people throughout the world. A glaring aspect of the pandemic is that not everybody’s lives (and health) have been affected equally. The health and economic costs of the crisis have fallen disproportionately on the most disadvantaged and vulnerable members of society, i.e., individuals involved in service work (so-called “frontline” occupations), as well as race-ethnic minority groups, particularly in the United States and the UK [1]. Social capital has been defined as the group of resources—for example, trust, norms, and the exercise of sanctions—available to members of a social group such as a neighborhood or a workplace. The salient feature of this approach is that social capital is conceptualized as a group attribute. In addition, social capital is a group of resources –for example, social support, information channels, social credentials—that are embedded within an individual’s social networks. In this approach, social capital is conceptualized as an individual attribute as well as a property of the group [2]. During times of crisis, resilient people are able to fall back on their social connections—i.e., their “social capital” —to weather adversity. Ironically, the public health response to COVID-19 has required social distancing measures that have handicapped people’s ability to mobilize social support.

During past crises and disasters, social capital has played a crucial role in the community’s response to emergencies [3,4,5]. Social capital is essential under these circumstances because it involves mutual support, social cohesion, cooperation, trust and resilience [6]. Networks are the conduit through which people access valuable information. For example, we hypothesized that during the early days of the pandemic crisis, cohesive communities were more effective in disseminating information about shelter-in-place orders, maintaining norms of avoiding high-risk activities (e.g., large social gatherings), and mobilizing collective action to assist members of the community in need. Understanding how individuals, households and the community access social capital during crises can provide new insight into ways of promoting preparedness [6].

At the same time, network connections can be “agnostic” with respect to outcomes, the so-called dark side of social capital, i.e., the resources exchanged within a group can be used for bad ends as well as good ends [7,8]. This involves the observation that social capital can entangle negative health outcomes and less desirable consequences. For example, the information that diffuses through a network can be damaging to public health (e.g., the spread of conspiracy theories regarding the coronavirus). Similarly, norms and beliefs that are propagated within a tightly knit group can be a threat to mounting an effective public health response, e.g., the anti-vaxxer movement. Communities with high social capital can also heighten hostility toward perceived outsiders, as witnessed by the rising incidence of anti-Asian attitudes (ranging from prejudice to hate crimes) throughout the world. Hence, it is important to understand the “dark side” of social capital to avoid the trap of presenting features of a community such as bounded solidarity and cooperation as the panacea to solve health-related problems.

Island nations, such as New Zealand, Taiwan, and Iceland, have been particularly successful in containing the pandemic. One obvious advantage is their natural geographic boundaries, enabling effective border controls. They also present a potential opportunity to study the role of social capital in responding to the threat of a pandemic. On the one hand, the geographic isolation of island nations and communities has meant that locals were forced to turn to each other for support during dire emergencies. This may account for their greater ability to mobilize cooperation from citizens, e.g., compliance with social distancing and mask wearing (“we are all in it together”). On the other hand, theory suggests that the same places may be more likely to express negative attitudes and mistrust toward outsiders during times of crisis—the dark side of bounded solidarity [9].

Menorca is a small island with 95,641 residents that thrives mostly on the tourism industry [10]. The local government closed the border during the strict lockdown phase, and travel to and from the island was restricted until the advanced recovery phases. The border closure lasted for more than 2 months. As of 5 April 2020, the island had 91 cases. However, the Government of the Balearic Islands acknowledged in their reports that only the most severe cases were detected at the beginning of the pandemic and that the true number was much higher [11]. At that time, the main hospital in Menorca did not have sufficient beds in its ICUs to care for all patients, and was forced to restrict outpatient care in order to open more ICU beds. Menorca has a Mediterranean culture. This involves several characteristics. Mediterranean societies are more familial in culture [12], and adult children in Mediterranean countries provide more support to parents than in other cultures such as the Nordic ones [13]. Family care prevails [14]. The way in which the relationship between the family group and its members manifests itself has implications for the way society functions. Young members of the family group set up households of their own or in a way in which families organize support for their most vulnerable members. A study in Spain found that the family network tends to be more extensive than the social network, which decreased with age due to health and functioning decline [15]. In fact, this relationship changes during the life course of individuals, and the social network becomes crucial when leaving the parental household.

We selected the island of Menorca as the setting of our study for several reasons. Menorcan society could be described as insular (e.g., relative mistrust of outsiders) and rooted in traditional Mediterranean culture, with strong systems of family and community social support. At the same time, the economy of the island depends heavily on tourism, thereby setting up potential tension between the imperative to keep economic activity alive even after the border closed versus obeying orders to shelter in place to protect the lives of vulnerable members in the community. This tension was further complicated by the drastic reduction in trust (of politicians and elites) following a recent economic crisis that affected the island especially severely. Thus, our project sought to understand the mechanisms through which social capital shaped the community response to the pandemic on the island of Menorca. Our specific aims were to understand emergent forms of social capital such as the creation of new networks and forms of solidarity that were not seen before the pandemic, as well as the so-called “dark side” of social capital, i.e., the negative consequences of social capital such as tensions between different groups and lack of trust during the emergency response to the first wave of the pandemic.

## 2. Materials and Methods

### 2.1. Sample, Timeline and Recruitment

**Figure 1:**
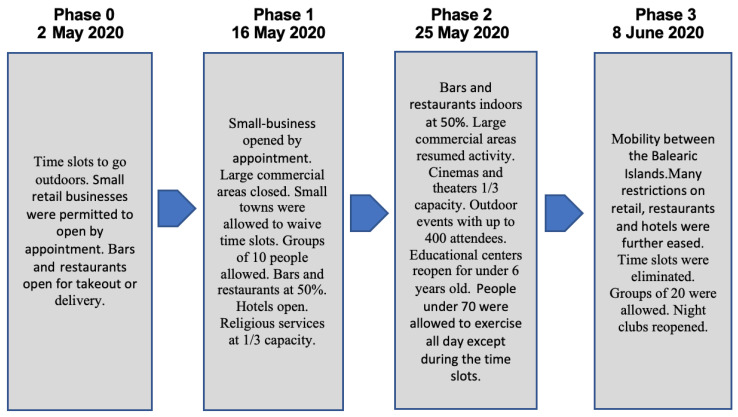
Emergency response and recovery phases: steps and characteristics.

The goal of sample recruitment was to interview residents of Menorca who had stayed on the island during the lockdown. The PI sent invitation letters to a convenience sample of 4 participants comprising residents of Menorca to initiate sample recruitment. From these, a snowball process was used to recruit more potential participants into the study. The four initial participants were not related, which allowed the study investigators to be connected to new participants belonging to different social groups. Potential participants who were unable to communicate or did not have a phone to be interviewed privately were not invited to participate. Out of 35 potential participants identified, 30 met the inclusion criteria (they had to be residents of Menorca and living in Menorca during the lockdown), and 25 agreed to be interviewed. Of the 25 participants interviewed, 14 were interviewed during the lockdown and 11 during the recovery phases of the lockdown. In Figure 1, we explain the features of the lockdown and the phases of recovery of the first wave of the pandemic implemented by the Government of Spain, and the dates on which Menorca started each phase. A more detailed description of each phase is available in the Appendix. This information is crucial to interpret the results of the study.

Participants in the study had a variety of age and job characteristics: occupations considered essential such as police officers, grocery store workers, and medical workers, and non-essential employees such as hospitality workers and participants that could telework). This allowed us to capture the diverse impact the public health crisis could have on people with such different profiles. In Table 1 we offer details about the demographic information of the sample. The age range of the participants was 26 to 89 years old, and 15 out of the 25 participants were female. We interviewed four hospital workers (two nurses, one physician and one lab technician), two grocery store cashiers, two restaurant owners, one pub owner, one pharmacist, two policemen, one software developer, one city hall official, the owner of an essential business, one banker, five people living in nursing homes and four retirees. All participants were permanent residents of the island. Of the 25 individuals, 21 were born on the island. The other four were born in mainland Spain, but had been on the island for more than 10 years. The Institutional Review Board at the University of Maryland Baltimore reviewed and approved this study.

### 2.2. Qualitative Study

We conducted qualitative telephone interviews (*n* = 25) between April and June of 2020 with permanent residents of Menorca, Spain. The PI (female) had been trained in qualitative research methods and conducted the interviews. The sample size was acceptable according to other qualitative studies [16,17,18,19,20], and managed to achieve saturation for the topics included in the interview guide. As Chamlee-Wright et al. describe in their work, qualitative approaches are particularly helpful in understanding questions of how people react to unprecedented situations in terms of emergent forms of social capital, dark sides of social capital and trust [5]. Furthermore, as these authors state in their work, signs of resilience and the ways in which individuals build their narratives for pandemic response and recovery efforts are particularly difficult to capture through surveys and quantitative analysis. The semi-structured interview format creates a window through which the researcher can see more clearly different forms of social capital. In this case, it was a way to access information from community members to frame their approach to pandemic response and recovery [5].

Our overall goal was to understand the mechanisms through which social capital shaped the community response to the pandemic on the island of Menorca, Spain, which was under a strict lockdown in 2020. Hence, the semi-structured interview guide had questions about pre-pandemic and pandemic emergency response and recovery events. Questions were developed to capture information on all the different indicators stated above (emergent forms of social capital, dark sides of social capital and trust). Examples of questions about social capital included the following: How frequently did you see your close friends before the lockdown imposed due to the COVID-19 outbreak? Are you looking after vulnerable people (older people who are socially isolated, people who are in nursing homes, etc.)? How has your community changed in terms of organization? Have you observed new forms of solidarity? Examples of questions on dark sides of social capital (lack of social cohesion or lack of trust): What do you think of people who are not permanent residents of the island and moved to Menorca from other parts of Spain/Europe after the COVID-19 outbreak? Do you trust politicians? Do you trust scientific leaders? During the interviews, the Government of Spain announced the end of the strict lockdown and the beginning of the recovery phases. Hence, in the interviews conducted during the recovery phases, we altered some questions to capture changes and explore new information. An example of a question asked during the recovery phase was: When you meet with family and friends, what kind of protective measures do you follow? Regarding protective measures, participants were asked to specify which measures they were following. If they stated they were not following the recommendations, participants were asked why they were not following the recommendations to identify the primary drivers of compliance. In addition, we explored emergent topics. The dynamic sampling strategy allowed us to observe if there was an evolution in the responses to the interview questions. However, since we did not follow up with individual participants, we can only make inferences about changes between individuals at different times and not changes within individuals at different times. In fact, 11 out of 25 participants were interviewed during the recovery phases. Hence, our strategy was to keep asking the same exact questions during the recovery phases, but adding new questions to understand the primary drivers of compliance. We concentrated on learning about the experiences of essential and non-essential workers and on having access to individuals of different ages. All conversations were audio recorded. The interview guide can be found in the Supplementary Materials.

### 2.3. Analysis

Audio recordings provide a useful check on the researcher’s memory, and provide a precise record of exactly how respondents frame problems, assess their situation, and craft their responses. By capturing the precise language of the narrative, the oral history is not only an account of the events that unfold; it is an account of the framework of thought that guides strategy formation [5]. This level of detail is difficult to absorb upon a first hearing (when the mind tends to focus on the basic sequence of events). Audio files, on the other hand, can be transcribed, and once transcribed, they can be systematically coded for patterns within and across interview transcripts. Hence, we used an integrated approach to guide the identification of codes and themes and select quotes across study participants [21]. First, a codebook was developed after a first revision of the content of the interviews. Second, the codebook was refined with the analysis of the interviews and used to code the materials using Atlas TI. We revised the content of the interviews and explored common themes, emergent themes and selected quotes to support each theme. Interview time ranged from 25 min to 55 min. When we analyzed the content of the interviews, we took into special consideration the time when the interview was conducted during the pandemic (strict lockdown or phases of recovery), sex, age and occupation of the respondents.

In the following section we describe the principal themes that were common across interviews and emergent themes. We organized the results section based on the different topics under investigation: bounded solidarity as an emergent form of social capital, ambivalence of island residents toward outsiders as a manifestation of the dark side of social capital originated during the pandemic, trust (in politicians, scientific leaders, the military, neighbors and co-workers) and lack of trust, and emergent topics (socioeconomic inequalities and intergenerational conflict). Therefore, the next section is organized by topic giving examples of quotes and informing about the following important characteristics of the respondents: sex, age, essential/non-essential workers/retired/in nursing homes and the time when the interview was conducted for better interpretation of the results. During the analysis, we concentrated on reaching saturation for the main topics under study: bounded solidarity as an emergent form of social capital, ambivalence of island residents toward outsiders as an example of the dark side of social capital originating during the pandemic, trust (in politicians, scientific leaders, the military, neighbors and co-workers) and lack of trust. In our study, we were able to reach saturation across participants despite their diversity and different profiles. For emergent topics, we did not prioritize reaching saturation, as we were focused on learning about new themes that were considered relevant for some of the participants of the study.

## 3. Results

### 3.1. Emergent Forms of Social Capital: Bounded Solidarity

Interview participants spoke highly of the new video calls with family and friends, a platform they had hardly used in the past (Table 2). Bounded solidarity was a common topic across all study participants, but it was emphasized by those participants that were interviewed during the strict lockdown. Two older adults (male resident of a nursing home and a retired female) remarked that they were always busy and were socially active before the lockdown and that all social routines suddenly changed. This was particularly challenging in older adults, since they had no other things to do during the lockdown.

“The nursing home has not changed anything. It all works the same with the same organization. I have changed some of my jobs—nursing home treasurer— and we cannot leave the nursing home. We used to go out a lot. My activities used to be bingo and dancing. We have a completely different life than the one we had before. I can only speak with my friends over the phone” (Male. 84. Nursing home resident. Interviewed during the lockdown).

“We used to have a lot of social life. I was into a lot of things. I used to go to university courses for seniors and with the “happy” grandmothers, to children’s story theater group, the book club and gym. Now nothing” (Female. 78. Retired. Interviewed during the lockdown).

Younger participants highlighted the parties in balconies that happened all over the country at 8 pm during the lockdown. At first, this was organized to thank medical staff for their work on the front line. The situation evolved in some neighborhoods where neighbors started to increase the time they were on the balconies and played bingo, danced, etc. For non-essential workers, this had been the only time in months they saw other people besides the ones in their household and people they encountered while doing essential shopping. The parties at 8 pm are a recurrent topic in the interviews.

“Suddenly we have met all our neighbors. Before this we didn’t know them. There are around nine houses on our street. We go out at 8 pm, and we have started to get to know each other. We’ve also had bingo at 7:30 pm with the children in the street. And other activities. Some people are very participative” (Female. 41. Pharmacist; interviewed during the lockdown).

“I have seen the town very empty. And a lot of people clapping after 8 pm. Today some people were pretending to get married wearing costumes, and they were joking from their balconies. At 1 pm. People were having a good time. And this kills time and makes people laugh. I saw a video of it because someone recorded it“ (Female. 61, market cashier; interviewed during Phase 0).

One participant really appreciated the support of her community. Her husband was hospitalized with COVID-19, and she was alone at home for weeks because she was not allowed to visit her husband at the hospital. Video calls with loved ones and expressions of support helped her during the disease of her husband.

“We have a video call with the family after 8 o’clock. I see my other daughter on the balcony. My children who live in Menorca have been able to go to the hospital and see their father who is hospitalized infected with COVID-19. I have had very bad days. Now I’m calm. We have a neighbor who usually makes a video during summer to celebrate summer festivities. This year he created a video with the neighbors of our village singing, dancing and playing drums. He included pictures of my husband in the hospital saying we should resist and fight against the virus“ (Female. 65. Retired. Husband hospitalized with COVID-19; interviewed during the lockdown).

Participants also cited new platforms to help each other. Residents of Menorca were able to mobilize collective action to assist members of the community, especially for those in need. The most relevant platforms were the development of an online social network where people could post messages when they had needs and others offered their support to cover these needs; the town hall had a registry of older adults living on their own and called them every week offering help and support; young people volunteered to be part of the Red Cross in case there was a need for additional staff; police officers received face masks and sanitizer from mainland Spain; a network of local businesses was launched to support the work of local people and a small village organized a community fundraiser.

“Yes, a social network for doing things for others has been created. If someone needed something, they posted it. This was mostly to help people who couldn’t go out (go to the pharmacy, market…). My father-in-law is 81 years old and has no problem using technology. Now there are people that have learnt a lot of stuff“ (Male. 48. Pub owner; interviewed during Phase 1).

“There has been a lot of promotion of local stores, and farmers have been selling produce online. And people have responded to those initiatives. I do believe there have been changes. Some people have been making masks and giving them away“ (Female. 47. Nurse; interviewed during the lockdown).

Two participants remarked on the difficulty they had with members of their family to keep them home. They highlighted that even though they were vulnerable to the pandemic, they went shopping every day. In fact, one participant pointed out that during the strict lockdown, older adults were the group that was always out compared to people of other ages. By contrast, other participants highlighted their efforts to maintain norms avoiding risky behaviors. For example, one participant pointed out that considering that her parents were over 70 and one of them was at high risk, she would delay all travel—even if it was allowed—to avoid infecting her parents. She pointed out that her brother and sister, who lived in Madrid, would not visit the island during summer to avoid exposing their parents to the virus. In short, we observed that a common topic was that participants were maintaining norms to avoid behaviors that were not recommended, but most of them mentioned knowing people that were not following the recommendations.

“My father has COPD and has not been out. I have helped my parents avoid any trips. Total different story with my in-laws, they haven’t been so obedient and have gone out more. In fact, a lot of elderly people in Ciutadella have not respected the stay-at-home orders and have been amongst the people that have gone out more. Especially at the beginning of the lockdown“ (Female. 41. Pharmacist; interviewed during the lockdown).

### 3.2. Dark Sides of Social Capital: Ambivalence of Island Residents toward Outsiders

We heard different opinions about opening the island to outsiders. On the one hand, almost all participants highlighted that tourists were considered a threat of infection. On the other hand, the islanders depend on them for tourist revenue. Hence, participants that economically depend on the tourism industry because they had a business such as a hotel, restaurant, pub, or shop were itching to reopen. Others with different types of jobs, including a medical doctor, who did not rely on tourism for their income, expressed the view that there was significant risk.

“With no health there is no economy. I understand. But with no economy there is no health, either. We would end up with no doctors, no nurses…“ (Male. 68. Business Owner. Interviewed during Phase 2).

“I am in favor of opening the island to tourism. But we need to test travelers and be able to control the situation. There should be a lot of investment in controls, but this is not happening. My father is at high risk since he is diabetic and has had heart attacks and pneumonia. I have brothers living in Madrid, and they will not come to Menorca for this reason“ (Female. 40. Online banking manager; interviewed during Phase 3).

### 3.3. Trust

One of the themes almost all participants agreed on was the lack of trust in politicians compared to trust in scientific leaders, the police and the military. Participants were particularly interested in talking about politicians and scientific leaders. When talking about politicians, some comments referred to their low quality, improvisation and changing strategies. Some participants expressed that they were willing to trust them but observed a lack of cohesion and coordination and that the problem of low quality politicians was shared with many other countries. Only one person pointed out that she trusted politicians. She thought the situation was totally new and acknowledged it was hard for politicians to deal with the pandemic.

“I don’t trust them. I’m not saying they did a bad job. But at the same time they didn’t do it right, because nobody was expecting this. On Monday they gave orders that were the last straw and they had to rectify. They said kids could go to the grocery store and other stuff. They screwed up. I’m not saying others would have done it better“ (Female. 41. Pharmacist; interviewed during the lockdown).

“I don’t trust them. It’s like having a lot of pigs together on a farm. First they fight, then they eat together and they end up rolling around in the same mud“ (Male. 89. Nursing home resident; interviewed during the lockdown).

Scientific leaders received more positive comments from all participants, albeit some of them were made with reservations. One participant pointed out that she trusted scientific leaders if they were not involved in politics, and others revealed doubts about this group. In short, some of them needed to trust some kind of authority, others thought scientists should take more into account the economy, and others pointed out the need to separate politicians from scientific leaders. In terms of trusting individuals, the sample was very divided, and there were different opinions. Some participants highlighted that they strongly trusted their neighbors and co-workers, whereas others underlined some negative experiences with them.

“I trust scientific leaders that are not involved in politics. If your salary comes from the state, you cannot tell the truth. And they are also hiding the truth from you“ (Female. 53. Lab technician; interviewed during the lockdown).

“I trust them. These people are very busy. But if they are not able to influence politicians to address the problem based on truth, it’s going to be very complicated. They don’t have the power to decide“ (Male. 48. Policeman; interviewed during Phase 0).

Lack of trust was a very important theme for almost all participants. This theme was common when talking about nonresidents moving to the island when the lockdown was announced. Basically, some participants pointed out that nonresidents were arriving from densely populated cities with many positive COVID-19 cases. One person talked about nonresidents infecting the island when they should have stayed were they usually live. Others acknowledged this had been a problem, but that they would have done the same if they lived in big cities, pointing out that big cities were the worst place to be in during a lockdown.

“The privileged got here. They are smart and use their power to get a better life. In Menorca we have privileges compared to other islands. The people that came here came to soil the island, they should have stayed home. People come here and they think they own the place“ (Female. 74, retired; interviewed during the lockdown).

“At first we really resented it. I hear that kind of opinion at the local store. They criticized the fact that people had come. My son was going to come and he didn’t come. It was too late. He wanted to come because he was starting a new job here. I disapprove of what some groups of people did. They should have come taking precautions. But they also spend money and that’s good. There should have been controls much earlier“ (Female. 61, market cashier; interviewed during Phase 0).

### 3.4. Emergent Themes: Socioeconomic Inequalities

Frontline workers vs. remote workers.

Interestingly, frontline workers—basically people working at hospitals and grocery stores—remarked that they were essential workers but did not complain about being on the front line. Other participants that were working remotely highlighted that they had been very lucky because they could telework.

“At the beginning it was overwhelming. There were long lines to access the shop. We had to remind people to keep their distances before accessing the business. Logistics were complicated“ (Female. 58. Supermarket cashier; interviewed during the lockdown).

“I am lucky to work in tech. We develop software. I can work from home. The company is very flexible“ (Male. 37. Software developer; interviewed during Phase 2).

### 3.5. Mediterranean Familial Culture

During the lockdown, only females highlighted that they were taking care of older adults. In addition, older adults pointed out that they were being taken cared of by their daughters. After the lockdown, the situation reversed. In the recovery phases, with schools still being closed, some participants pointed out that they relied on their families as the resource to take care of their kids. Two female participants explained that they were taking care of their grandchildren. The participants who reported being caregivers were all female in our sample. A common comment was that people were very relaxed and were not following the recommendations of physical distancing with family members. This was the case with friends as well.

“My daughter, who works at a bank, stops by and asks if we need anything. She goes shopping and to the pharmacy for us“ (Female. 78. Retired; interviewed during the lockdown).

“The person who helped me the most was my daughter, who is here next door. She goes shopping. I don’t have a driver’s license. She takes me to the hospital to see my husband. She is healthy and does absolutely everything“ (Female. 65. Retired. Husband hospitalized with COVID-19; interviewed during lockdown).

### 3.6. Inter-Generational Conflicts

We observed that some participants talked about younger generations with resentment. Students moved back to the island during the lockdown, and there were some complaints about their behaviors, e.g., not taking preventive measures and restrictions seriously and possibly spreading the virus. However, at the same time, most of them said it was OK, since they were from the island.

“I guess there are all sorts of cases. A lot of students arrived. At first nobody liked it, but you understand the psychological part of wanting to be with the family. But it could have been done better. The people from Madrid were the first to arrive, because schools, universities, etc. shut down earlier than in other places and they just turned it into holidays. The problem is that they started leaving, leaving, leaving right from the start. You could see them everywhere. All the people that were able to leave and spread it all over Spain“ (Female. 41. Pharmacist; interviewed during the lockdown).

“Very irresponsible. I even think if your son or daughter is studying away from home and comes back, it’s very irresponsible. They have spread the virus to other places. A lot of people didn’t respect the 15-day quarantine“ (Female. 39. Policewoman, interviewed during Phase 0).

## 4. Discussion

It is evident from the findings that social capital is an important resource with the capacity to organize help and support. Residents of Menorca relied upon existing social structures and emergent forms of social capital to overcome the hardships during lockdown. However, the dark sides of social capital, with lack of social cohesion, also emerged as an important negative issue. We identified sources of tension that were not resolved, as well as important sociodemographic differences that are primary drivers for health inequalities.

In the introduction, we pointed out that a pandemic is not only a physical hazard, but also a social one. A pandemic gives us clues to understand where a society is vulnerable and where it is resilient. In Menorca, COVID-19 was a severe “stress test” for social capital, and more specifically, for social cohesion. On the one hand, we observed important forms of social cohesion, with participants highlighting several forms of collective action and solidarity to help citizens at risk. There were testimonies of participants understanding and making efforts to maintaining norms to protect citizens at risk, especially older adults [6]. However, we observed tensions and contradictions between different groups that suggested a fraying of social cohesion and sociodemographic differences. The first tensions we observed were between those in favor of locking down the island versus those that needed to make a living from tourism. This pitted the interests of business owners, who needed to keep the island open to make their business profitable, against the interests of the vulnerable (e.g., service workers in the tourist industry). Second, we identified tensions from older generations to younger generations. In the interviews, the young were considered an important source of viral spread at the beginning of the lockdown when many students moved from mainland Spain to the island. At the same time, we believe that young people felt relatively “immune” from the serious effects of infection during the early COVID-19 outbreak, and suffered more from lockdown fatigue. In addition, we think there may be resentment in younger generations because their student life and their jobs have been sacrificed in order to protect the old. These particular tensions emphasize the need to reinforce bridging social capital, the connections between individuals that are dissimilar, e.g., in different age groups [22]. Whereas there was strong bonding social capital between older adults that felt the threat of the virus and between youth that felt their student life and jobs were sacrificed, there was a lack of bridging the social capital that is key to help build trust and maintain channels of communication between these “feuding” groups. Third, we identified tensions between those that are more individualistic and prioritize their own interests versus individuals that prioritize society as a whole. This happened between participants that economically depended on the tourism industry and others that had a different type of job. Despite the fact that we observed several forms of solidarity during the interviews, with participants complying with the lockdown in the interest of the rest of society, after the lockdown some people no longer wanted to follow the recommendations and were reluctant to sacrifice their social life. Fourth, we observed tensions between insiders versus outsiders. Menorca displayed bounded solidarity against an external threat with cooperation among members. However, there was hostility toward outsiders. Outsiders brought danger and the threat of infection to islanders. Fifth, there were tensions between those who were able to telework versus those who were forced to continue going to work. There was an important difference between the participants that were on the front line (medical and pharmacy staff, police officers, supermarket cashiers, and owners of businesses that were considered essential) and those that were not. These participants talked about experiences that could not be compared with participants that were not on the front line. While those on the front lines talked about job organization and safety, non-essential workers remarked on missing family and friends, their strategies to protect vulnerable populations, the difficulties some of them were experiencing while working from home with kids, how lucky they felt because they were allowed to telework, and the effects of lockdown fatigue. Finally, we witnessed tensions between the need to trust authorities (e.g., scientists) versus distrust (of politicians). This was a common topic in all the interviews we performed. In the past, in the midst of situations such as the economic crisis that started in 2008, there was a notable decline in trust in institutions, together with an increase in social trust [23]. An increase in social trust involves more civic participation [24,25], which is very positive. However, the lack of trust in institutions is an indicator of an important deficit of the entire society that can have negative consequences [26]. Levels of trust during a crisis such as a pandemic usually change. Trust in politicians and leaders depends on the coherence of the crisis response. When citizens witness a disorganized government response that includes frequent changes of recommendations, trust decreases [23]. The lack of trust in politicians was critical in the interviews with almost all participants, whereas trust in scientific leaders was better. Indeed, many participants highlighted that they trusted scientific leaders if there were a disagreement between them and politicians.

Social cohesion was compromised in all situations mentioned above. Many of these tensions were not resolved after the first wave of the pandemic and remained a problem in the second wave. In fact, the island opened to tourism after the first wave, but tourists did not arrive in large numbers and the economy was highly impacted. The problem was that the number of COVID-19 cases increased after the opening of the island, and youth became the center of contagion. Hence, the tensions between the old and youth were even worse after the data collection of this study. Furthermore, Christmas is an important holiday in Spain. Families and friends get together, often in big groups, and people socialize. The month before Christmas, we witnessed a major debate about how to celebrate the festivities. Again, tensions arose between those with a more individualistic approach versus those that were thinking about society as a whole. Some expressed the impulse to act in their own interest and meet with all family members whereas others acted following the interests of the rest of society, limiting the number of people in social gatherings to help preserve public health. Overall, policies in Spain prioritized protecting public health, often against the interests of business owners. However, this was not always the case. Differences existed between regions, and some political parties chose to prioritize the economy over public health. In addition, when the incidence of COVID-19 stabilized but was still high, preventive measures were relaxed, a move that many public health experts considered premature and capable of harming not only public health, but also the economy in the long term. In Spain, science did not become as politicized as in the United States, but there was widespread confusion and distrust of expert advice. Some political parties fomented doubts about groups of experts and expert advice. This can have very serious consequences for population adherence to preventive measures and acceptance of a vaccine.

Spain’s response to the pandemic and its consequences needs to be evaluated considering that the country has a quasi-federal political system, with 17 autonomous regions, each with its own government. Public health functions are decentralized and therefore are in the hands of the regional governments. In 2011, the government passed a public health bill that required the creation of a Spanish Public Health Agency that would act as a coordinator for all the different regional agencies. By 2020, Spain had still not created the national Public Health Agency. So, in order to overcome the problems of a decentralized system and centralize the public health powers of all territories, Spain declared a state of emergency. Political decisions were made based on the recommendations of the Health Emergencies Coordination Center, a public agency in charge of coordinating the epidemiological response of the autonomous regions. In fact, they make recommendations to the government and to the CISNS, the National Health System’s Interterritorial Council. We suggest that the decentralization of Spain and the lack of a Spanish Public Health Agency played an important role in the pandemic response and its consequences [27]. Citizens remarked on the lack of consensus between the central government and some regional governments, especially those governed by parties on the opposite side of the political spectrum. The lack of support and cohesion of some political parties and the perception of last-minute decisions exacerbated the problem of lack of trust. Bargain and Aminjonov demonstrated that societies with high levels of social trust during COVID-19 significantly decreased their mobility related to non-essential activities compared to societies with low social trust [28], and Woelfert and Kunst suggested that social trust was associated with lower social distancing tendencies among participants with low levels of political trust [29]. As a consequence, social trust has become polarized. Some parts of the population may have higher levels of social trust than before, after observing solidarity and emergent forms of social capital. Others, after observing that people were breaking the rules during the lockdown and going back to normal too fast without following the rules during the recovery phases, may have harmed it. This is a subject that needs to be further investigated, since the scenario we are in is different from previous scenarios and this pattern may be different than in previous crises.

Our study has limitations. We did not follow up with participants and we cannot evaluate changes within individuals. However, an important strength is that we used a dynamic sampling strategy that allowed us to evaluate the evolution of the participants’ responses during the evolving pandemic situation. Another limitation is that the interviews were conducted over the phone, not face-to-face, which limits the interpretation of the responses. A third limitation is that more females than males agreed to be part of our study, and our findings should be interpreted taking into account that not enough males were interviewed. In addition, we have interpreted and formed conclusions based on not only the main topics under study, but also emergent themes. As we specify in the methods section of this manuscript, it is important to note that information collected under emergent themes was purely exploratory and was endorsed by some participants only, not the majority. Finally, we did not interview non-residents of Menorca who moved to the island before the lockdown. This limited our capacity to evaluate trust and mistrust and we could not fully evaluate and arrive at conclusions about the dark sides of social capital in this matter. However, an important strength of our study is that we were able to recruit participants with a variety of occupations and ages.

## 5. Conclusions

We believe that the analysis presented here points the way toward a larger discussion about the sociodemographic factors that make participants especially vulnerable, the role of social capital guiding pandemic response and recovery, and the need to promote social cohesion to solve frictions between groups. It was clear that in the first wave of the pandemic, emergency and recovery efforts came from within the social networks that provided different types of support, cohesion and cooperation. However, we observed tensions and confrontations between different groups that were not solved, showing a lack of social cohesion. Given the important role of social resources, it is pertinent to ask what should be done in terms of future outbreaks and pandemic response. The central government should establish a line of funding for local authorities to articulate the full use of existing social capital in recovery by maintaining social networks in recovery processes, reinforcing social cohesion, and disseminating information about preventive measures and civic behavior. The investment in social networks and social capital is a long-term need that should consider sociodemographic vulnerability. In fact, the promotion of social cohesion between different population groups is a key component to solve frictions between different groups.

## Figures and Tables

**Table 1:** Sample characteristics (*n* = 25).

Age	Gender	Occupation	Time Interview
26	Female	Nurse	Strict lockdown
58	Female	Supermarket cashier	Strict lockdown
53	Female	Lab technician	Strict lockdown
84	Male	Nursing home resident	Strict lockdown
47	Female	Nurse	Strict lockdown
84	Female	Nursing home resident	Strict lockdown
89	Male	Nursing home resident	Strict lockdown
65	Female	Retired with husband hospitalized with COVID-19	Strict lockdown
78	Female	Retired living with a couple	Strict lockdown
78	Female	Nursing home resident	Strict lockdown
78	Female	Nursing home resident	Strict lockdown
74	Female	Retired living with a couple	Strict lockdown
41	Female	Pharmacist	Lockdown with flexibility
84	Male	Retired living alone	Lockdown with flexibility
61	Female	Market cashier	Phase 0
48	Male	Policeman	Phase 0
39	Female	Policewoman	Phase 0
48	Male	Pub owner	Phase 1
54	Male	Business owner	Phase 1
37	Male	Software developer	Phase 2
61	Female	City Hall official	Phase 2
68	Male	Business owner	Phase 2
62	Male	Restaurant owner	Phase 3
40	Female	Online banking manager	Phase 3
43	Male	Medical doctor	Phase 3

**Table 2:** Topics under investigation with themes and quotes derived from the interviews (*n* = 25).

Topics Related to Social Capital		Examples of Quotes
Emerging forms of social capital	Bounded solidarity	“The nursing home has not changed anything. It all works the same with the same organization. I have changed some of my jobs –nursing home treasurer- and we cannot leave the nursing home. We used to go out a lot. My activities used to be bingo and dancing. We have a completely different life than the one we had before. I can only speak with my friends over the phone” (Male. 84. Nursing home resident). “We used to have a lot of social life. I was into a lot of things. I used to go to university courses for seniors and with the “happy” grandmothers, to children’s story theater group, the book club and gym. Now nothing” (Female. 78. Retired). “There is a lot of partying and excitement on the street at 8 pm when we thank the medical staff from our window, balcony and terraces. It’s an emotional moment” (Female. 53. Lab technician). “We speak with our family very often. We always use video calling. Something we never did with friends from Menorca. With family, video calling has become a continuum. Grandma is an expert of social media now. Now with some friends we do crafts together by live zooming once a week. Something we could have done before” (Female. 47. Nurse). “My daughter, who works at a bank, stops by and asks if we need anything. She goes shopping and to her pharmacy for us” (Female. 78. Retired). “We have a video call with the family after 8 o’clock. I see my other daughter on the balcony. My children who live in Menorca have been able to go to the hospital and see their father who is hospitalized infected with COVID-19. I have had very bad days. Now I’m calm. We have a neighbor who usually makes a video during summer to celebrate summer festivities. This year he created a video with the neighbors of our village singing, dancing and playing drums. He included pictures of my husband in the hospital saying we should resist and fight against the virus“ (Female. 65. Retired. Husband hospitalized with COVID-19). “Yes, a social network for doing things for others has been created. If someone needed something he/she posted it. This was mostly to help people who couldn’t go out (go to their pharmacy, market…). My father-in-law is 81 years old and has no problem using technology. Now there are people that have learnt a lot of stuff“ (Male. 48. Bar Pub owner). “Suddenly we have met all our neighbors. Before this we didn’t know them. There are around 9 houses on our street. We go out at 8 pm and we have started to get to know each other. We’ve also had bingo at 7:30 pm with the children in the street. And other activities. Some people are very participative” (Female. 41. Pharmacist). “I have seen the town very empty. And a lot of people clapping after 8 pm. Today some people were pretending to get married wearing costumes and they were joking from their balconies. At 1 pm. People were having a good time. And this kills time and makes people laugh. I saw a video of it because someone recorded it” (Female. 61, market cashier). “There has been a lot of promotion of local stores and farmers have been selling produce online. And people have responded to those initiatives. I do believe there have been changes. Some people have been making masks and giving them away” (Female. 47. Nurse). “Now he (her son) comes to see me through the window and we speak over the phone. They have given us resources to make video calls. My granddaughter can call me. I have grandchildren in Girona and Barcelona and I have been able to see them. This has changed everything. Before we had never done it” (Female. 84. Nursing home resident). “I haven’t been out since the lockdown started on March 14th. My situation is good because the town hall called me during the first week to ask if I needed anything. I’m fortunate because I have four children and I am very well taken care of. I follow the situation on TV. Now I make video calls and that way I can see everybody every day. All my children” (Male. 84. Retired). “The face masks have been the most relevant issue. We have received masks from mainland Spain, that is an example of solidarity. Even retired ladies from all over Spain have been sending them. They have also sent us hand sanitizer. Solidarity with us too. The 8 pm walks. Some drawings they sent us go straight to the heart” (Male. 48. Policeman). “Phone and Skype. We live very close. Grandma died this week and we were able to go and see her, but we were not able to see her before. I have video calls with my mother. A lot of people have called to offer their help to those in need. As law enforcement officers, we just told them that it was already organized and that it wasn’t allowed. There are more volunteers at the Red Cross than ever before” (Female. 39. Policewoman). “My father has COPD and has not been out. I have helped my parents to avoid any trips. Total different story with my in-laws, they haven’t been so obedient and have gone out more. In fact, a lot of elderly people in Ciutadella have not respected the stay-at-home orders and have been amongst the people that have gone out more. Especially at the beginning of the lockdown” (Female. 41. Pharmacist). “I am part of the Association of Restaurants of Menorca. We held meetings to see how we could contribute to different local governments. We have done webinars and teleconferences for training, to solve questions, and so on” (Male. 54. Business owner). “Our small village organized a community fundraiser. Shoes factories stopped their production to produce masks and hospital gowns” (Female. 61. City Hall official).
Dark side of social capital	Ambivalence of island residents toward outsiders	“I am in favor of opening the island to tourism. But we need to test travelers and be able to control the situation. There should be a lot of investment in controls, but this is not happening. My father is at high risk since he is diabetic and has had heart attacks and pneumonia. I have brothers living in Madrid and they will not come to Menorca for this reason” (Female. 40. Online banking manager). “All tourists should come with PCR done two days before flying. If not, they shouldn’t be allowed to fly. Right now Menorca is very vulnerable” (Male. 43. Medical Doctor). “The privileged got here. They are smart and use their power to get a better life. In Menorca we have privileges compared to the other islands. The people that came here came to soil the island, they should have stayed home. People come here and they think they own the place” (Female. 74. retired). “I would prefer to keep the island closed to tourists this summer. But of course, I don’t have a business. My family doesn’t depend on tourism” (Male. 37. Software developer). “With no health there is no economy. I understand. But with no economy there is no health, either. We would end up with no doctors, no nurses…” (Male. 68. Business Owner. Interviewed during Phase 2). “People that came here should have taken preventive measures. It is true we need them to come because they spend money here. But the arrival of visitors has not been controlled” (Female. 61, market cashier). “There is economic pressure on health” (Female. 61. City Hall official). “Unfortunately, we have an economy based on tourism. Now, I am afraid that if things are not done properly and there is a resurgence of the pandemic, most businesses will be on bankruptcy” (Male. 62. Restaurant owner).
Trust	Trust in politicians	“I don’t trust politicians. They are the worst scum. I am apolitical” (Female. 74, retired). “I don’t trust them. Some people see the situation earning 100,000 euros, while we see it with an empty pocket” (Male. 84. Nursing home resident). “I don’t trust them. I’m not saying they did a bad job. But at the same time, they didn’t do it right, because nobody was expecting this. On Monday they gave orders that were the last straw, and they had to rectify. They said kids could go to the grocery store and other stuff. They screwed up. I’m not saying others would have done it better” (Female. 41. Pharmacist). “I don’t trust them. It’s like having a lot of pigs together on a farm. First they fight, then they eat together and they end up rolling around in the same mud” (Male. 89. Nursing home resident). “No, they are all the same. They didn’t build a team to help each other. They have just fought each other. They are not politicians, they are nothing. This situation will get a lot of politicians out of the way, after this they will have to go back home” (Male. 84. Retired). “Not anymore. At first I did. Now I can’t see them well-balanced. There are too many people wanting to call the shots” (Female. 61, market cashier). “I want to trust them. Maybe too much. Everyone wants to do things and go forward. That is the problem, and they are not coordinated” (Female. 84. Nursing home resident). “I don’t trust them, I see a lot of improvisation. And they change their minds with social media” (Male. 48. Policeman). “The quality is low and it’s happening in all countries. In Spain we could fire practically all of them. One can’t see people that are capable of doing things right. Two weeks ago, the Labor Minister said that bars would not be able to reopen until New Year’s Eve. Eight Months closed? I can go and die” (Male. 48. Pub owner).
Trust	Trust in scientific leaders	“I trust scientific leaders that are not involved to politics. If your salary comes from the state, you cannot tell the truth. And they are also hiding the truth from you” (Female. 53. Lab technician). “Yes, I do trust them. It is because of them that the outcome will be good. It is an honor for them” (Female. 84. Nursing home resident). “I want to trust them. We need to trust someone. But I don’t trust them completely” (Female. 58. Supermarket cashier). “I trust them. These people are very busy. But if they are not able to influence politicians to address the problem based on truth, it’s going to be very complicated. They don’t have the power to decide” (Male. 48. Policeman). “Yes, I do trust them. But I have my reservations about them. I believe that sometimes the decisions they make as scientists should be made taking into account what is best for the economy. To increase their decision power, sometimes their decisions are too close to the people they favor. If politicians are not skilled enough to interpret things right, they don’t make good decisions” (Male. 48. Pub owner). “Yes. But unfortunately we are a country that cuts back on research and the best scientists we have end up working in countries where they have a lot more help to develop their work. I have relatives that are University Professors who, 15 years ago, had teams of 15 people working on very promising projects. All these projects stopped and could not be finalized” (Male. 62. Restaurant owner).
	Trust in the military	“I don’t trust them, they are just another arm of the politicians” (Female. 47. Nurse). “I do, my husband was in the military. They have done a lot of work disinfecting” (Female. 65. Retired. Husband hospitalized with COVID-19).
	Trust in the neighbors and co-workers	“I don’t trust them. I’ve had a problem with one of them. During the lockdown, she waited (referring to her neighbor) for me in our parking garage and she coughed on me on purpose, and she was not wearing a mask. I recorded everything. I recorded it and I reported her to the police. We’ll see what happens with our lawyers. I’m not a person that lives on the staircase” (Female. 53. Lab technician). “There are all sorts of people (“talking about neighbors”). Some of them have just ignored the lockdown” (Male. 48. Pub owner). “I only trust my co-workers at the lab. I don’t know about the rest of the staff at the hospital. I have to fight to get the samples in the right conditions. The problem is that people from outside the lab don’t provide the samples in the right conditions and I have to argue with them so they do it right. I have to decontaminate the samples. It could be done better” (Female. 53. Lab technician).
Emergent themes: socioeconomic differences	Frontline workers versus being allowed to work from home	“At the beginning it was overwhelming. There were long lines to access the shop. We had to remind people to keep their distances before accessing the business. Logistics were complicated” (Female. 58. Supermarket cashier). “I am lucky to work in tech. We develop software. I can work from home. The company is very flexible” (Male. 37. Software developer).
	Mediterranean familial culture	My daughter, who works at a bank, stops by and asks if we need anything. She goes shopping and to her pharmacy for us (Female. 78. Retired). “The person who helped me the most was my daughter who is here next door. She goes shopping. I don’t have a driver’s license. She takes me to the hospital to see my husband. She is healthy and does absolutely everything” (Female. 65. Retired. Husband hospitalized with COVID-19). “Older adults are the ones who have been most affected. I don’t take care of older adults now. I used to take care of my granddaughter. Now I can’t. But I will do it again after the lockdown” (Female. 58. Supermarket cashier). “My mother was very worried. She is 86 years old. She worked a lot in the garden. She received phone calls from the City Council. My daughter took care of everything. Now with my grandson we do not take any preventive measure. I wore a mask on the first day I saw him after the lockdown. The other days we have had a normal life” (Female. 61. City Hall official).
	Inter-generational conflicts	“I guess there are all sorts of cases. A lot of students arrived. At first nobody liked it, but you understand the psychological part of wanting to be with the family. But it could have been done better. The people from Madrid were the first to arrive, because schools, universities, etc. shut down earlier than in other places and they just turned it into holidays. The problem is that they started leaving, leaving, leaving right from the start. You could see them everywhere. All the people that were able to leave and spread it all over Spain” (Female. 41. Pharmacist). “I have mixed feelings. It’s very wrong that they came to Menorca. But I also have friends that are studying away from home that came back before everything shut down. I understand them. But they contaminated us. It was wrong” (Female. 26. Nurse. Ex-COVID-19). “Honestly, I don’t think it was right. The students that were in Barcelona or in Madrid wanted to return home. They might be OK. But not the other ones” (Female. 65. Retired. Husband hospitalized with COVID-19). “Very irresponsible. I even think if your son or daughter is studying away from home and comes back, it’s very irresponsible. They have spread the virus to other places. A lot of people didn’t respect the 15-day quarantine” (Female. 39. Policewoman).

## Data Availability

Transcriptions of the interviews are available upon request.

## Supplementary Materials

The following are available online at https://www.mdpi.com/article/10.3390/ijerph182312720/s1, Document S1: Supplementary material.
