# Supplementary material for: Social Capital during the First Wave of the COVID-19 Outbreak: The Case of the Island of Menorca
Source: Int J Environ Res Public Health. 2021 Dec 2;18(23):12720. doi: 10.3390/ijerph182312720 (PMC8656712; doi:10.3390/ijerph182312720)
Supplement: Supplementary file 1 [file ijerph-18-12720-s001.zip › ijerph-1413584-supplementary.pdf]

Supplementary material: Description of the lockdown and the recovery phases.

The lockdown only allowed residents to go to work if they were health care personnel or workers of businesses considered essential, such as grocery stores, pharmacies or public transport. In addition, adults could only leave their homes to shop for groceries, to go to the pharmacy or to go to the doctor. Children were not allowed to leave the house and people were not allowed to exercise outdoors. The lockdown started on March 16th. On April 26th, children under 14 were allowed to play outside their homes for an hour from 9 am to 9 pm with an adult. Only groups of one parent/guardian and up to three children were allowed. The recovery stage had four phases. Phase 0 started in Menorca on May 2nd and involved preparing for the transition to "relief" measures such as the possibility of working out outdoors from 6 to 10 am and from 8 to 11 pm. Older adults and highly-dependent adults were allowed outside their homes from 10 am to 12 pm and from 7 to 8 pm. Children were allowed outside their homes for an hour from 12 to 7 pm. Small retail businesses were permitted to open by appointment only and bars and restaurants were allowed to open for takeout or delivery service only. The preparation of all public spaces was intensified with signs reminding the public of social distancing and hygiene measures. Two weeks later, Menorca moved on to Phase 1, and small-business activities partially restarted without appointments required. Large commercial areas remained closed. During Phase 1, towns with a population under 5,000 were allowed to waive the time slots by age for outdoor activities. Groups were limited to 10 people. Bars and restaurants were allowed to open for outdoor service only at 50% capacity. In addition, hotels started to operate again with the exclusion of common areas. There was a preferential time slot for outdoor activities for people over 70. Religious services also restarted at one-third of their capacity. The use of masks was highly recommended, especially on public transport. Phase 2 started on May 25th. It began with the reopening of indoor service at bars and restaurants at 50% of their capacity. Large commercial areas resumed their activity with limitations and groups of up to 15 people were allowed. Cinemas and theaters opened with pre-assigned seats at one-third occupancy. And outdoor events were allowed with up to 400 seated attendees. In addition, educational centers for children under 6 years of age were allowed to reopen for students whose parents were required to go back to the workplace. People under 70 were allowed to exercise all day except during the time slots reserved for senior citizens -10 am-12 pm and 7 pm-8 pm. Phase 3, the final stage of the recovery plan before the 'new normal', started in Menorca on June 8th. It allowed mobility between the Balearic Islands and many restrictions on retail, restaurants and hotels were further eased. Time slots were eliminated and groups of up to 20 people were allowed, public transport resumed at full capacity and night clubs were reopened with limitations [29].

Supplementary material:

How frequently did you see your close friends before being in lockdown due to the COVID-19 outbreak?

How frequently did you see your family members before being in lockdown due to the COVID-19 outbreak?

How have you continued these relationships with close friends and family? How frequently do you speak with them now and which platform do you use to communicate? How do you support each other now?

Probe: Make sure we get information about how participants continue staying in touch with neighbors, providing mutual social support, seeking the most updated information about the COVID-19 pandemic, etc.

Probe: Get information on possible virtual parties, etc.

Are you looking after the most vulnerable members? (Older people who are socially isolated, people who are in nursing homes, etc.)

How has your community changed in terms of organization? Have you observed new forms of solidarity? Please specify.

Have you been working during the COVID-19 outbreak? Are you considered essential personnel? How has your employer organized work conditions during the COVID-19 outbreak?

How is this situation impacting the monthly income of your family? Have you had the need to look for financial assistance or do you plan to look for financial assistance?

Do you feel anxiety, depression or any other mental health issues? Have you had the need to look for help?

Do you trust politicians? Do you trust scientific leaders? Do you trust the police? Do you trust the military? Do you trust your neighbors? Do you trust your co-workers?

What do you think of people who are not primary residents of the island who decided to move to the island from mainland Spain or other parts of Europe after the COVID-19 outbreak?
